# Using Consumer-Grade Physical Activity Trackers to Measure Frailty Transitions in Older Critical Care Survivors: Exploratory Observational Study

**DOI:** 10.2196/19859

**Published:** 2021-02-23

**Authors:** Ben Kim, Miranda Hunt, John Muscedere, David M Maslove, Joon Lee

**Affiliations:** 1 School of Public Health and Health Systems University of Waterloo Waterloo, ON Canada; 2 Department of Critical Care Medicine Queen's University Kingston, ON Canada; 3 Data Intelligence for Health Lab Cumming School of Medicine University of Calgary Calgary, AB Canada; 4 Department of Community Health Sciences Cumming School of Medicine University of Calgary Calgary, AB Canada; 5 Department of Cardiac Sciences Cumming School of Medicine University of Calgary Calgary, AB Canada

**Keywords:** frailty, frail elderly, wearable electronic devices, fitness trackers, activity trackers, heart rate, sleep monitoring, critical care outcomes

## Abstract

**Background:**

Critical illness has been suggested as a sentinel event for frailty development in at-risk older adults. Frail critical illness survivors are affected by increased adverse health outcomes, but monitoring the recovery after intensive care unit (ICU) discharge is challenging. Clinicians and funders of health care systems envision an increased role of wearable devices in monitoring clinically relevant measures, as sensor technology is advancing rapidly. The use of wearable devices has also generated great interest among older patients, and they are the fastest growing group of consumer-grade wearable device users. Recent research studies indicate that consumer-grade wearable devices offer the possibility of measuring frailty.

**Objective:**

This study aims to examine the data collected from wearable devices for the progression of frailty among critical illness survivors.

**Methods:**

An observational study was conducted with 12 older survivors of critical illness from Kingston General Hospital in Canada. Frailty was measured using the Clinical Frailty Scale (CFS) at ICU admission, hospital discharge, and 4-week follow-up. A wearable device was worn between hospital discharge and 4-week follow-up. The wearable device collected data on step count, physical activity, sleep, and heart rate (HR). Patient assessments were reviewed, including the severity of illness, cognition level, delirium, activities of daily living, and comorbidity.

**Results:**

The CFS scores increased significantly following critical illness compared with the pre-ICU frailty level (*P=*.02; *d*=−0.53). Survivors who were frail over the 4-week follow-up period had significantly lower daily step counts than survivors who were not frail (*P=*.02; *d*=1.81). There was no difference in sleep and HR measures. Daily step count was strongly correlated with the CFS at 4-week follow-up (*r*=−0.72; *P=*.04). The average HR was strongly correlated with the CFS at hospital discharge (*r*=−0.72; *P=*.046). The HR SD was strongly correlated (*r*=0.78; *P*=.02) with the change in CFS from ICU admission to 4-week follow-up. No association was found between the CFS and sleep measures. The pattern of increasing step count over the 4-week follow-up period was correlated with worsening of frailty (*r*=.62; *P=*.03).

**Conclusions:**

This study demonstrated an association between frailty and data generated from a consumer-grade wearable device. Daily step count and HR showed a strong association with the frailty progression of the survivors of critical illness over time. Understanding this association could unlock a new avenue for clinicians to monitor and identify a vulnerable subset of the older adult population that might benefit from an early intervention.

## Introduction

### Frailty Among Critical Illness Survivors

Frailty is a state of increased vulnerability to adverse health outcomes due to the loss of physiological and cognitive reserves [1]. Although frailty often overlaps with terms such as disability and comorbidity, it has been well described that frailty is an independent concept that can be quantitatively separated [2]. Frailty is recognized as a dynamic state, and recent studies have highlighted the need to quantify changes between the stages of frailty to better inform clinicians with the development of tailored treatments [3].

Critical illness has been suggested as a sentinel event for the development of frailty, especially for at-risk older adults [4]. Frailty is frequently evaluated as a prognostic tool in critical care settings to better guide decision making by clinicians and to manage the expectations of patients and families on health outcomes [5]. Critical illness survivors who were frail before the illness, in comparison with those who were not frail, have a significantly higher mortality rate [5-7], are more likely to acquire functional dependence [5,8], have lower quality of life [5], and are more frequently rehospitalized within 12 months [7]. However, no studies have examined the progression of frailty throughout and beyond critical illnesses and how physical and functional recovery is related to changes in frailty.

### Wearable Device Uses in Research

Older adults are the fastest growing group of consumer-grade wearable device users [9]. The potential uses of these devices for general wellness and clinical purposes have gathered the interest of many stakeholders, including patients, care providers, funders, governments and policy makers, and technology developers [10-14]. The opportunity to leverage data generated from such devices for clinical and medical purposes is expected to increase as these devices are becoming smaller, cheaper, and ever more accessible in the recent years [15]. Coupled with the logistic and financial challenges of monitoring critical illness survivors’ functional recovery outside the hospital setting, many recent research studies investigated several possible uses, including wearable devices as a tool to objectively measure the physical activity level [16,17], sedentary behaviors [18], and mobility [19] and to screen for frailty [20].

### Objectives

We examined the data generated from wearable devices for their association with the progression of frailty after hospital discharge and hypothesized significant associations between frailty and physical activity, sleep quality, and heart rate (HR), as reported by the wearable devices. In particular, we hypothesized that survivors who are frail would have lower physical activity, diminished sleep quality, and impaired HR control compared with those who are not frail. We also hypothesized that survivors whose frailty returns to the precritical illness level would have a higher physical activity level, better sleep quality, and tighter HR control than those who have a persistent increase in frailty after hospital discharge.

## Methods

### Study Design and Settings

This observational study was conducted at Kingston General Hospital in Kingston, Ontario, Canada. Patients were recruited from the FORECAST (Frailty, Outcomes, Recovery and Care Steps of Critically Ill Patients) study, which assessed an array of clinical measurements and frailty. For this study, patients were recruited during their admission to the intensive care unit (ICU) from July 2017 to August 2018. Participants were followed up at 4 weeks after hospital discharge.

A convenience sampling method was used to recruit patients aged 55 years and older. They were included in this study if they lived within the city or close by to ensure feasibility of attending the 4-week follow-up session. Patients were excluded if shared decision makers were not available to collect collateral history. We also excluded patients who had medical conditions that might have interfered with the proper use of the wearable devices, including those admitted to the ICU with catastrophic neurological illness that was not likely to be altered by ICU care (eg, massive stroke requiring ICU care, spinal cord injury with neurological deficit), those diagnosed with primary neuromuscular pathology or atrial fibrillation, or those dependent on a wheelchair for mobility. Patients with nonsinus rhythms were excluded. Patients were further excluded if they had an expected survival of less than 1 month.

### Data Collection and Instrumentation

#### Determination of Frailty

Frailty was assessed using the Clinical Frailty Scale (CFS), a tool that has been widely used in critical care settings [1]. The CFS has been shown to outperform other frailty assessment tools in the geriatric population in correctly differentiating major health outcomes such as hospital admission and fall incidents [21]. It is especially suitable for older adults and critically ill populations who may lack cognitive or physical capabilities to answer and perform necessary tasks to be assessed for frailty with other tools [1]. The CFS ranges from 1 to 9, where 1 denotes very fit and 9 represents terminally ill. CFS scores of 1 to 3 are considered not frail, a score of 4 is considered prefrail, and 5 or higher is considered frail. However, a CFS score of 4 or higher was considered frail in this study. Frailty was assessed by one of the 3 experienced research coordinators available at a given time.

#### Wearable Device

Fitbit Charge HR (Fitbit; hereafter referred to as Fitbit) is a commercially available wearable device worn on the wrist. It uses a triaxial accelerometer to measure motion. These data are used to estimate physical activity, sedentariness, and sleep quality. Fitbit also measures the changes in elevation using an altimeter. Fitbit uses an optical HR sensor (ie, photoplethysmography) to measure the HR between 30 and 220 beats per minute.

In this study, we collected physical activity levels including daily step count, active time, and sedentary time. Fitbit automatically deems the active time when a physical activity of at least three metabolic equivalents is performed. Sleep-related information is generated, including total time in bed, total sleep time (TST), awake time, and awake count. Sleep efficiency was calculated as the percentage of sleep time over the TST. Sleep time was determined by subtracting the awake time from the TST. HR was measured every minute. HR data were used to assess the average daily HR, SD of average daily HR, and average nocturnal HR. The average nocturnal HR was calculated using only the HR recorded during sleep as classified by the TST.

#### Other Variables

The research coordinators reviewed the patients’ medical charts and collected demographic information, including age, sex, height, and weight. The degree of comorbidity and the ability to perform activities of daily living were calculated using the Charlson Comorbidity Index [22] and the Katz index [23], respectively. The severity of illness and delirium were collected and calculated using the Acute Physiology and Chronic Health Evaluation II (APACHE II) [24] and the Confusion Assessment Method-ICU [25], respectively. The major critical care treatments received during the ICU stay, including invasive mechanical ventilation, noninvasive ventilation, vasopressor use, corticosteroid use, continuous renal replacement therapy, and intermittent hemodialysis, were collected. The ICU length of stay (ICU LOS) and hospital LOS were calculated from chart reviews.

### Procedure

**Figure 1:**
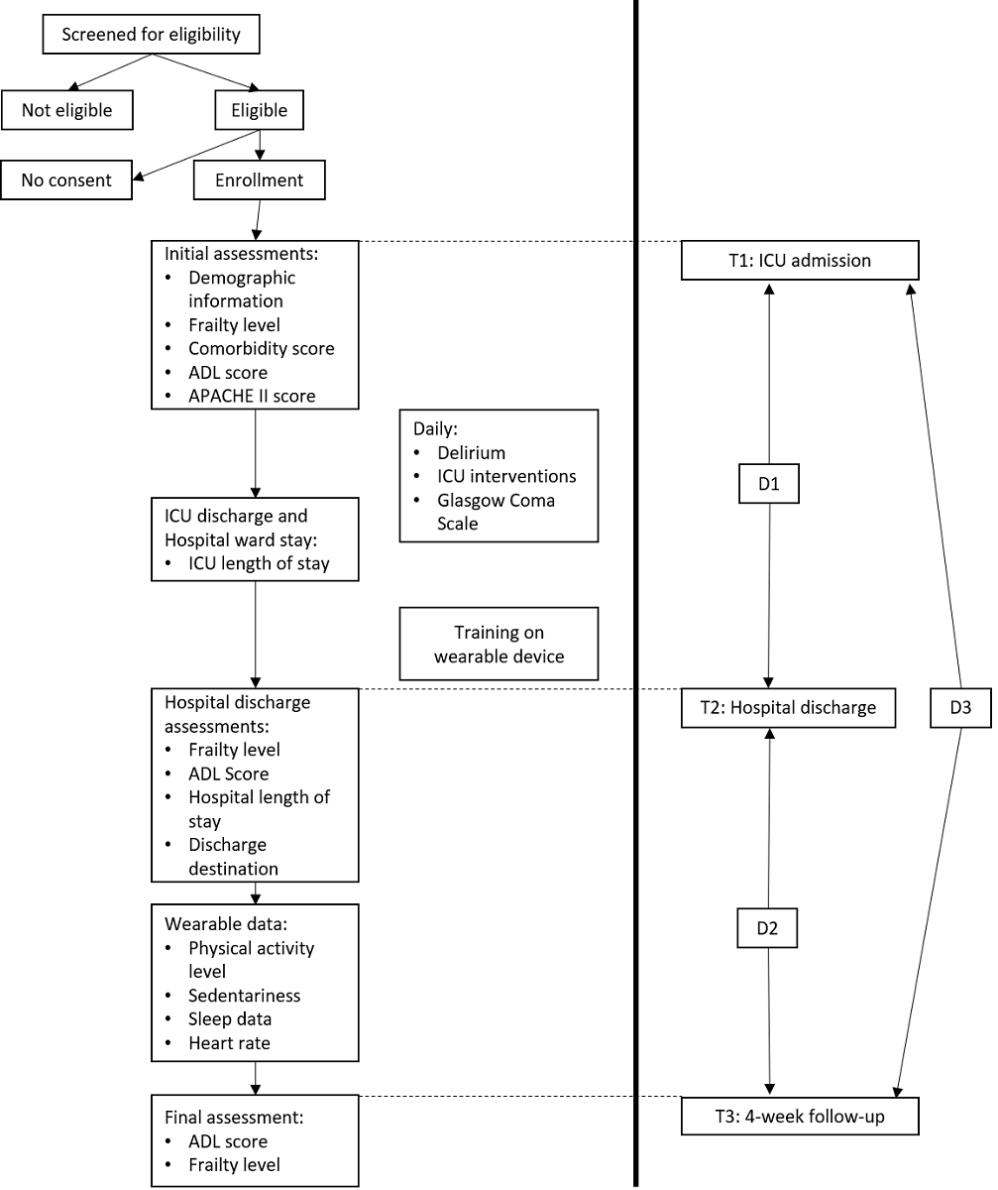
Study procedure and time points of assessment and measurement tools. ADL: Activities of Daily Living; APACHE: Acute Physiologic Assessment and Chronic Health Evaluation; ICU: intensive care unit.

A total of 3 trained research coordinators interviewed the patients at 3 different time points: ICU admission (T1), hospital discharge (T2), and 4-week follow-up (T3). The assessment conducted at T1 was used to establish the baseline information (ie, pre-ICU admission). Figure 1 outlines the study procedure and time points for the assessments and measurement tools. All participants received a wearable device at ICU discharge and were trained on its use during the hospital ward stay before hospital discharge. Participants were encouraged to wear the device during the ward stay, but only the posthospital discharge data were used for analyses. The time between T1 and T2 is referred to as D1, between T2 and T3 as D2, and between T1 and T3 as D3, hereafter.

### Data Analyses and Interpretation

Descriptive statistics and univariable comparisons of means, medians, and proportions were performed to describe the demographic information and patient characteristics according to frailty status. The Shapiro-Wilk normality test was performed to check for normality. Student *t* test, Mann-Whitney *U* test, or chi-square test was performed to check for independence between frail and nonfrail survivors at T3. Cohen *d* was used to evaluate the effect size when a statistically significant difference was found.

The Pearson correlation coefficient and Spearman rank correlation coefficient were calculated to analyze the correlation between the data collected from the wearables and the changes in the CFS score over D1, D2, and D3. Their relationships with patient demographics and medical data were further examined.

A linear regression was performed for individual patients’ daily step count, daily TST, daily awake duration, and HR over D2. The slope of the regression line (hereafter referred to as the slope) was examined for its relationship with the changes in the CFS score over D1, D2, and D3 by performing Spearman rank correlation analysis. Patients with fewer than 5 days of wearable data were excluded from this analysis.

Statistical significance was set at α=.05 for all statistical results. Statistical analysis was performed using R Studio (R version 3.6.0, R Studio version 1.2.1335, R Studio Inc).

### Ethics, Consent, and Permissions

This study was approved by the office of research ethics at the University of Waterloo (ORE22219) and the Queen’s University Health Sciences and Affiliated Teaching Hospitals Research Ethics (ROMEO/TRAQ 6020644).

## Results

### Recruitment

A total of 16 patients admitted to the ICU were recruited after they provided informed consent between July 2017 and August 2018. Overall, 2 patients withdrew from the study, and 2 patients’ data were lost because of technical issues. In total, we had 12 patients with wearable device data collected successfully (Table 1).

**Table 1:** Baseline characteristics, frailty, disability, and comorbidity scores.

Patient characteristics		Frail at T3^a^	Nonfrail at T3^b^	*P* value
**Demographics**				
	Patients, n	7 (58.33)	5 (41.67)	N/A^c^
	Age (years), mean (SD)	66 (8.12)	67.8 (5.07)	.81
	Sex (female), n (%)	6 (85.71)	1 (20.00)	.02^d^
	BMI, kg m^−^^2^	30.22 (8.36)	26.34 (16.01)	.21
**Type of admission**				
	Medical, n (%)	1 (8.33)	10 (83.33)	N/A^c^
	Surgical, n (%)	0 (0.00)	1 (8.33)	N/A
Intensive care unit length of stay (days), mean (SD)		15.29 (5.19)	13.40 (9.63)	.67
Hospital length of stay (days), mean (SD)		24.57 (10.49)	20.60 (16.32)	.62
Acute Physiology and Chronic Health Evaluation II score		26.71 (6.63)	29.00 (2.45)	.81
Glasgow Coma Scale score		7.43 (4.54)	5.20 (2.28)	.56
Charlson Comorbidity Index score		1.57 (2.07)	1.00 (1.22)	.21
Katz at T1^a^ score		5.00 (1.29)	5.60 (0.55)	.59
Katz at T3^b^ score		5.71 (0.76)	6.00 (0.00)	.50

^a^ICU admission.

^b^4-week follow-up.

^c^N/A: not applicable, as *P* value cannot be computed.

^d^*P*<.05.

The patients were aged between 55 and 77 years, with a mean age of 66.75 (SD 6.80) years, and 7 patients were female. There were significantly more frail female participants than male participants (*P*=.02). The mean ICU LOS was 14.50 (SD 7.03) days and hospital LOS was 22.92 (SD 12.69) days. The mean APACHE II score at T1 was 27.67 (SD 5.25). Overall, 7 of the 12 patients were classified as frail at T3. There were no other major differences in baseline characteristics between frail and nonfrail patients at T3.

### Clinical Frailty

**Figure 2:**
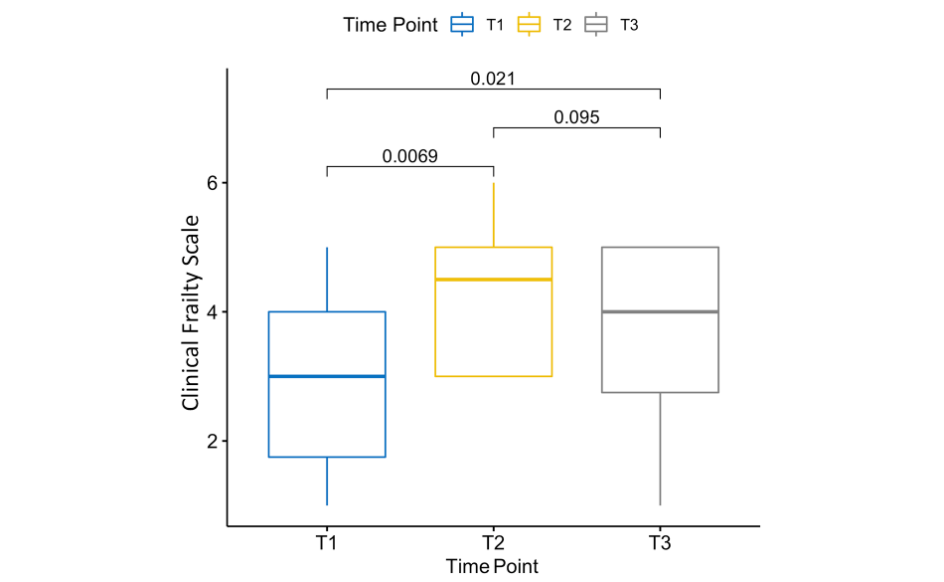
A boxplot of the Clinical Frailty Scale score at T1: ICU admission, T2: hospital discharge, and T3: 4-week follow-up (n=12). ICU: intensive care unit.

Critical illness had a profound effect on the patient’s frailty level (Figure 2). Compared with the baseline CFS score at T1, the CFS score at T2 increased significantly (*P*=.007; *d*=−1.13). A general trend of improvement in frailty level was observed over D2; however, the difference was not statistically significant (*P=*.10; *d*=0.59). At T3, the frailty level returned to that at the baseline for 6 patients, whereas it worsened for 6 patients. Overall, the CFS score increased significantly over D3 (*P=*.02; *d*=−0.53). The changes in frailty level at different time points are summarized in Table 2.

**Table 2:** Changes in Clinical Frailty Scale score between ICU admission and 4-week follow-up.

Frailty changes	ICU^a^ admission to hospital discharge, n	Hospital discharge to 4-week follow-up, n	ICU admission to 4-week follow-up, n
Improved	0	2	0
No change	5	4	6
Worsened	7	6	6

^a^ICU: intensive care unit.

### Frailty and Wearables Data

Of the 12 patients, 3 wore the wearable devices for fewer than 5 days over D3 (Table 3). On average, patients wore the wearables for 26.33 days. Frail patients at T3 had significantly lower daily step counts than nonfrail patients (1336.40 vs 3781.04 steps; *P=*.02; *d*=1.81). They engaged in lesser daily physical activity than their counterparts (2.02 vs 16.34 minutes per day; *P=*.04; *d*=0.94). There was no difference in sleep and HR measures between the frail and nonfrail groups.

**Table 3:** Data collected from the wearable devices (n=9).

Wearable device measures			Frail at T3^a^		Nonfrail at T3^b^		*P* value
Patients, n (%)			5 (56)		4 (44)		N/A^c^
Days worn, mean (SD)			30.20 (8.73)		21.50 (8.27)		.17
**Physical activity variables**							
	Daily step count, mean (SD)	1336.40 (1091.07)		3781.04 (1389.37)		.02^d^	
	Sedentary time (min per day), mean (SD)	84.11 (55.75)		104.95 (49.78)		.58	
	Active duration (min per day), mean (SD)	2.02 (3.83)		16.34 (10.66)		.04^d^	
**Sleep measures**							
	Total sleep time (min per night), mean (SD)	419.71 (166.62)		336.25 (134.07)		.81	
	Total time in bed (min per night), mean (SD)	456.31 (182.49)		362.16 (144.88)		.84	
	Awake time (min per night), mean (SD)	24.427 (11.20)		21.30 (11.33)		.69	
	Awake count (times per night), mean (SD)	1.65 (0.62)		1.54 (1.10)		.85	
	Sleep efficiency (%), mean (SD)	91.72 (2.35)		92.70 (2.00)		.53	
**HR^e^ measures**							
	Average HR (bpm^f^), mean (SD)	86.93 (7.10)		80.38 (13.18)		.38	
	Heart rate standard deviation, bpm (SD)	8.81 (1.97)		10.66 (3.16)		.32	
	Average nocturnal HR, bpm (SD)	86.42 (5.87)		74.10 (20.27)		.27	

^a^T1: Intensive care unit admission.

^b^T3: 4-week follow-up.

^c^N/A: not applicable.

^d^*P*<.05.

^e^HR: heart rate.

^f^bpm: beats per minute.

The correlations between the wearable device data and frailty are summarized in Table 4. Daily step count strongly correlated with the baseline CFS at T1 (*r*=−0.76; *P=*.03) and the CFS score at T3 (*r*=−0.72; *P=*.006). Sedentary time strongly correlated with the CFS score at T1 but did not reach statistical significance (*r*=−0.66; *P=*.07). The average HR strongly correlated (*r*=−0.72; *P=*.046) with the CFS score at T2, and HR SD also strongly correlated (*r*=0.78; *P=*.02) with the CFS change over D3. No relationship was found between sleep measures and CFS scores. No patient characteristics had a significant relationship with the CFS score (see Multimedia Appendix 1 for the exact *P* values for each correlation coefficient).

**Table 4:** Correlations between the data collected from the wearables and the frailty level and its change overtime (n=9).

Wearable device measures and patient characteristics			Correlation (*r*)										
			Frailty at T1^a^		Frailty at T2^b^		Frailty at T3^c^		Frailty change over D1^d^		Frailty change over D2^e^		Frailty change over D3^f^
**Physical activity data**													
	Daily step count	−0.76^g^		−0.35		−0.72^g^		0.55		−0.46		0.14	
	Active time	−0.62		0.03		−0.53		0.63		−0.56		0.18	
	Sedentary time	−0.66		−0.39		−0.53		0.41		−0.24		0.25	
**Sleep data**													
	In bed	0.10		0.13		0.42		−0.01		0.32		0.45	
	Total sleep time	0.08		0.13		0.40		0.01		0.31		0.46	
	Awake time	−0.26		−0.07		0.07		0.22		0.13		0.50	
	Awake count	−0.31		0.06		−0.10		0.37		−0.15		0.33	
	Sleep efficiency	0.23		−0.10		0.12		−0.32		0.20		−0.19	
**HR^h^ data**													
	Average HR	−0.24		−0.72^g^		−0.16		−0.28		0.37		0.13	
	Heart rate standard deviation	−0.55		−0.05		−0.05		0.54		−0.01		0.78^g^	
	Average nocturnal HR	0.06		−0.21		−0.19		−0.22		−0.04		−0.37	
**Patient characteristics**													
	Age	0.18		0.56		<0.01		0.24		−0.45		−0.27	
	BMI	0.42		0.38		0.47		−0.11		0.15		0.04	
	Intensive care unit length of stay	−0.01		0.21		<0.01		0.17		−0.17		0.02	
	Hospital length of stay	0.15		0.15		0.05		−0.03		−0.07		−0.14	
	Charlson Comorbidity Index	0.56		0.12		0.29		−0.44		0.19		−0.44	
	Glasgow Coma Scale	0.24		−0.06		0.15		−0.27		0.19		−0.16	
	Changes in activities of daily living	0.06		0.34		0.05		0.20		−0.23		−0.02	
	Acute Physiology and Chronic Health Evaluation II	0.19		0.47		−0.12		0.17		−0.50		−0.47	

^a^T1: Intensive care unit admission.

^b^T2: Hospital discharge.

^c^T3: 4-week follow-up.

^d^D1: Intensive care unit admission to hospital discharge.

^e^D2: Hospital discharge to 4-week follow-up.

^f^D3: Intensive care unit admission to 4-week follow-up.

^g^*P*<.05.

^h^HR: heart rate.

### Frailty and Wearable Data Trends Over Time

**Figure 3:**
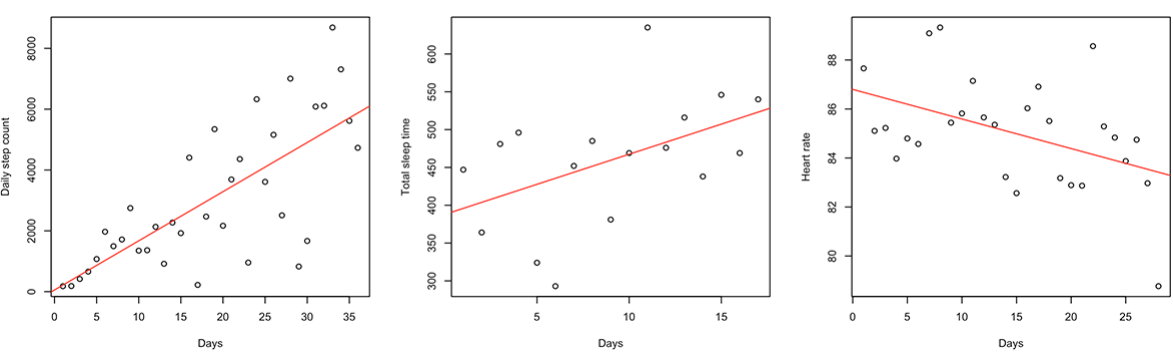
Example of the slope of linear regression line for daily step count, total sleep time, and heart rate. The slope of linear regression line represents the changes over over hospital discharge to 4-week follow-up.

The slope of the linear regression line for daily step count, TST, and HR was calculated to investigate the relationship between frailty and wearable device data trends over time (Figure 3). The slope of the daily step count demonstrated strong correlations with the CFS change over D1 (*r*=0.71; *P=*.01) and D3 (*r*=0.65; *P=*.03) (Table 5). The slope of HR strongly correlated with frailty change over D3 (*r*=0.62; *P=*.03).

**Table 5:** Correlation between the slope of daily step count, total sleep time, heart rate and the Clinical Frailty Scale (CFS) scores at intensive care unit admission, hospital discharge, and 4-week follow-up and changes in CFS over intensive care unit admission to hospital discharge, hospital discharge to 4-week follow-up, and intensive care unit admission to 4-week follow-up.

Time points and frame	Slope					
	Step count (*r*)	*P* value	Total sleep time (*r*)	*P* value	Heart rate (*r*)	*P* value
CFS^a^ score at T1^b^	−0.55^c^	.08	0.59^c^	.07	−0.31	.32
CFS score at T2^d^	0.27	.43	0.12	.74	−0.12	.72
CFS score at T3^e^	−0.18	.60	0.32	.36	0.10	.75
CFS change over D1^f^	0.71^g^	.01	−0.49	.15	0.21	.52
CFS change over D2^h^	−0.38	.25	0.21	.56	0.20	.54
CFS change over D3^i^	0.65^g^	.03	−0.52	.13	0.62^d^	.03

^a^CFS: Clinical Frailty Scale.

^b^T1: intensive care unit admission.

^c^*P*<.10.

^d^T2: Hospital discharge.

^e^T3: 4-week follow-up.

^f^D1: Intensive care unit admission to hospital discharge.

^g^*P*<.05.

^h^D2: Hospital discharge to 4-week follow-up.

^i^D3: Intensive care unit admission to 4-week follow-up.

## Discussion

### Frailty Transitions Following Critical Illness

In this exploratory observational study, we observed 12 older adult survivors of critical illness from the time of their admission to the ICU until 4-weeks after their hospital discharge. Physical recovery was monitored using a wearable device. Frailty was assessed at multiple time points throughout their ICU and hospital stay and at 4 weeks after discharge. A total of 6 patients became frailer after their critical illness, whereas the frailty of the other 6 returned to their precritical illness levels. No participant’s frailty improved above their pre-ICU baseline state. The incidence rate of worsening frailty over 3 years is reported to be approximately 0.6% and 1.3% for healthy men and women, respectively [26]. A noticeably higher incidence rate in the study sample confirms the notion that critical illness is a triggering event in the transition to a frail state [4].

### Patterns of Wearable Measures and Frailty Transitions

We demonstrated the association between a lower physical activity level and increased frailty level. This was evident from a significantly lower daily step count and active time by the frail survivors compared with their nonfrail counterparts. This finding is consistent with a previous study that used a wearable device worn on the upper arm and reported a significantly reduced step count by frail survivors compared with a healthy control group [16]. Our results suggest that the rate at which an individual increases daily step count following critical illness may be an important indicator for the recovery of frailty back to the precritical illness level. Those whose frailty worsened showed a significantly higher rate of increase in their daily step counts (*P*=.03). We initially suspected that the magnitude of the positive slope was amplified because of lower step counts among those whose frailty worsened. However, the average step count was not significantly different between those whose frailty worsened and those whose frailty did not change (*P=*.63). We further speculated that the difference in baseline frailty level may contribute to this finding; however, there was no significant difference in baseline frailty between the 2 groups (*P=*.49). Another possible explanation may be an increase in frailty because of nonphysical characteristics such as impaired cognitive function. Future research should confirm this relationship and investigate possible explanations. Understanding this relationship may help clinicians to accurately identify patients who will benefit from strengthened transitional care.

The pattern of increasing HR and the SD of HR were shown to be related to the worsening of frailty following critical illness. These findings are in line with the theoretical understanding of frailty as a concept of impaired homeostasis [27]. These patterns may be caused by the inability to evoke dynamic physiological processes to restore equilibrium. Studies that examined HR variability have concluded that frailty is associated with impaired cardiac autonomic control [28,29]. However, empirical evidence for the relationship between HR and frailty is lacking. Increased resting HR was found to be associated with functional decline among older adults [30], increased inflammatory markers [31], and an increased mortality rate among trauma patients [32].

To the best of our knowledge, this is the first study to investigate frailty by collecting and analyzing longitudinal HR data from a consumer-grade wearable device. The use of consumer-grade wearable devices to monitor HR has garnered the interest of many researchers in recent years. Its feasibility and accuracy have been researched in different populations, including patients who are critically ill [33]. Many studies have demonstrated its feasibility and acceptable compliance level, but its capacity to measure HR accurately has been questioned, especially for the detection of nonsinus rhythms such as tachycardia and bradycardia [33,34]. Despite this, our study used longitudinal HR data to successfully uncover the association among frailty, HR, and its SD. Future studies should expand on this relationship and its potential use as a screening and monitoring tool for frailty and the detection of early signs of clinical deterioration among critical illness survivors.

Poor sleep quality, particularly nighttime disturbances, was reported to be associated with an increased risk of frailty among community-dwelling older adults [35,36]. Frequently perturbed sleep in hospitals adversely affects patient’s recovery [37]. However, we found no significant association between sleep measures and changes in frailty. This may be explained by the inaccurate measures of sleep quality using wearable devices. The exact model of the device used in this study has been validated against polysomnography (PSG) for healthy adolescents and the same device brand among young adults [38,39]. However, it was noted that the performance of these devices may be poor in populations with low sleep quality or a high number of motionless wake episodes. Continued efforts to use consumer-grade wearable devices for routine sleep monitoring should be encouraged because the current methods such as PSG and sleep journals are not feasible because of their high cost and inaccuracy among patients who are critically ill [33].

### Implications for Consumer-Grade Wearable and Frailty Research in Critical Care Setting

Survivors of critical illness are uniquely situated as their physiological and cognitive reserves (ie, frailty) have been pushed to their limit and beyond. The successful recovery of frailty back to the precritical illness level is crucial for protection from subsequent critical illness. Unsuccessful recovery of frailty places an individual in a vulnerable state in which a lesser illness may lead to amplified adverse health outcomes, thereby requiring greater health care resources [40,41]. Our study demonstrated the possibility of early detection of unsuccessful frailty recovery in the first 4 weeks of post-ICU discharge using a wearable device. Identifying such a vulnerable subset of critical illness survivors warrants the timely delivery of frailty interventional programs that have been shown to improve frailty as well as various functional capabilities for community-dwelling older adults [42]. Furthermore, wearable devices have the potential to enhance the monitoring of physical activities in ecological settings, which can guide clinicians and researchers further by complementing the supervised data acquired in traditional settings [43].

### Limitations

Our study has several limitations. The exploratory nature of the study resulted in a restrictive sample size from a single ICU that is not representative of the entire critical ill population. It limited the generalizability of the findings to other populations. Other research studies reported significant differences in the age between frail and nonfrail patients, but our study sample did not. The small sample size prevented us from stratifying patients into nonfrail, at-risk, and frail groups. The addition of another level of frailty may have helped us interpret the slope of linear regression in more detail for daily step count, sleep time, and HR. Furthermore, patients who were critically ill were discharged from the ICU to a hospital ward before being discharged, which led to varied hospital ward LOS. This may have affected the assessment of frailty at the 4-week follow-up session. However, the hospital ward LOS was not significantly different between patients who are frail and not frail (13.4 vs 7.0 days; *P*=.20). We chose a 4-week follow-up to investigate the early recovery process immediately following the critical illness. A longer observation period of critical illness survivors will benefit future studies as full functional and physical recovery is achieved over 6- to 12-month periods for 25% to 50% of older critical illness survivors [44,45].

### Conclusions

In this study, we observed the physical recovery of critical illness survivors using a wearable device. Unsuccessful recovery of frailty to precritical illness level at 4 weeks after hospital discharge was related to a significantly lower step count followed by a high rate of increase in step count. This unsuccessful recovery was also related to an increase in HR over the same period. Sleep measures did not correlate with frailty. Our study demonstrated the possibility of using consumer-grade wearables as a tool to understand frailty progression for survivors of critical illness. We also demonstrated the added value of longitudinal wearable data. Consumer-grade wearables evolve rapidly, and future research should focus on leveraging new features such as electrocardiogram and more accurate measures of physical activity, sleep, and HR.

## Appendix

Multimedia Appendix 1 Correlations between the data collected from the wearables and the frailty level and its change overtime with exact P values.

## Abbreviations

APACHE II: Acute Physiology and Chronic Health Evaluation II
CFS: Clinical Frailty Scale
HR: heart rate
ICU: intensive care unit
ICU LOS: intensive care unit length of stay
PSG: polysomnography
TST: total sleep time
