# Supplementary material for: Using Consumer-Grade Physical Activity Trackers to Measure Frailty Transitions in Older Critical Care Survivors: Exploratory Observational Study
Source: JMIR Aging. 2021 Feb 23;4(1):e19859. doi: 10.2196/19859 (PMC8081159; doi:10.2196/19859)
Supplement: Multimedia Appendix 1 [file aging_v4i1e19859_app1.docx]

Multimedia Appendix 1. Correlations between the data collected from the wearables and the frailty level and its change overtime with exact p values.

|  | Frailty at T1 | (*P*-value) | Frailty at T2 (*P*-value) | (*P*-value) | Frailty at T3 (*P*-value) | (*P*-value) |
| --- | --- | --- | --- | --- | --- | --- |
| Physical activity data |  |  |  |  |  |  |
| Daily step count | -0.76* | 0.02 | -0.35 | .11 | -0.72* | .006 |
| Active time, minutes per day | -0.62 | 0.12 | 0.03 | .43 | -0.53 | .061 |
| Sedentary time, minutes per day | -0.66+ | 0.06 | -0.39 | .36 | -0.53 | 0.13 |
| Sleep data |  |  |  |  |  |  |
| In bed, minutes per night | 0.10 | 0.42 | 0.13 | .75 | 0.42 | 0.53 |
| Total sleep time, minutes per night | 0.08 | 0.43 | 0.13 | .74 | 0.40 | 0.53 |
| Awake time, minutes per night | -0.26 | 0.76 | -0.07 | .78 | 0.07 | 0.76 |
| Awake count, times per night | -0.31 | 0.91 | 0.06 | .23 | -0.10 | 0.74 |
| Sleep quality | 0.23 | 0.46 | -0.10 | .50 | 0.12 | 0.27 |
| Heart rate data |  |  |  |  |  |  |
| Average heart rate | -0.24 | 0.91 | -0.72* | .046 | -0.16 | 0.94 |
| Heart rate SD | -0.55 | 0.88 | -0.05 | .30 | -0.05 | 0.99 |
| Average nocturnal heart rate | 0.06 | 0.62 | -0.21 | .81 | -0.19 | 0.99 |
|  |  |  |  |  |  |  |
| Patient characteristics |  |  |  |  |  |  |
| Age | 0.18 | 0.61 | 0.56+ | .06 | <0.01 | 0.99 |
| Sex, female | 0.50 | 0.45 | -0.20 | .53 | 0.76* | 0.004 |
| BMI | 0.42 | 0.481 | 0.38 | .23 | 0.47 | 0.12 |
| ICU length of stay | -0.01 | 0.409 | 0.21 | .51 | 0.00 | 0.99 |
| Hospital length of stay | 0.15 | 0.183 | 0.15 | .64 | 0.05 | 0.87 |
| Charlson comorbidity index | 0.56 | 0.206 | 0.12 | 0.706 | 0.29 | 0.36 |
| Glasgow Coma Scale | 0.24 | 0.155 | -0.06 | 0.848 | 0.15 | 0.65 |
| Changes in ADL | 0.06 | 0.772 | 0.34 | 0.279 | 0.05 | 0.89 |
| APACHE II score | 0.19 | 0.636 | 0.47 | 0.123 | -0.12 | 0.71 |

|  | Frailty change over D1 (*P*-value) |  | Frailty change over D2 (*P*-value) |  | Frailty change over D3 (*P*-value) |  |
| --- | --- | --- | --- | --- | --- | --- |
| Physical activity data |  |  |  |  |  |  |
| Daily step count | 0.55 | .13 | -0.46 | .23 | 0.14 | .57 |
| Active time, minutes per day | 0.63* | .56 | -0.56 | .32 | 0.18 | .97 |
| Sedentary time, minutes per day | 0.41 | .20 | -0.24 | .38 | 0.25 | .98 |
| Sleep data |  |  |  |  |  |  |
| In bed, minutes per night | -0.01 | .14 | 0.32 | .99 | 0.45 | .88 |
| Total sleep time, minutes per night | 0.01 | .16 | 0.31 | .97 | 0.46 | .89 |
| Awake time, minutes per night | 0.22 | .64 | 0.13 | .69 | 0.50 | .94 |
| Awake count, times per night | 0.37 | .79 | -0.15 | .75 | 0.33 | .27 |
| Sleep quality | -0.32 | .35 | 0.20 | .52 | -0.19 | .40 |
| Heart rate data |  |  |  |  |  |  |
| Average heart rate | -0.28 | .32 | 0.37 | .11 | 0.13 | .47 |
| Heart rate SD | 0.54 | .57 | -0.01 | .89 | 0.78* | .02 |
| Average nocturnal heart rate | -0.22 | .43 | -0.04 | .26 | -0.37 | .73 |
|  |  |  |  |  |  |  |
| Patient characteristics |  |  |  |  |  |  |
| Age | 0.24 | .48 | -0.45 | .16 | -0.27 | .55 |
| Sex, female | -0.62* | .009 | 0.90* | <.001 | 0.32 | .60 |
| BMI | -0.11 | .85 | 0.15 | .27 | 0.04 | .71 |
| ICU length of stay | 0.17 | .56 | -0.17 | .61 | 0.02 | .88 |
| Hospital length of stay | -0.03 | .95 | -0.07 | .87 | -0.14 | .62 |
| Charlson comorbidity index | -0.44 | .15 | 0.19+ | .48 | -0.44+ | .09 |
| Glasgow Coma Scale | -0.27 | .83 | 0.19 | .24 | -0.16 | .57 |
| Changes in ADL | 0.20 | .21 | -0.23 | .52 | -0.02 | .84 |
| APACHE II score | 0.17 | .32 | -0.50 | .10 | -0.47 | .08 |
